# On the use of flexible excess hazard regression models for describing long-term breast cancer survival: a case-study using population-based cancer registry data

**DOI:** 10.1186/s12885-019-5304-2

**Published:** 2019-01-28

**Authors:** R. Schaffar, A. Belot, B. Rachet, L. Woods

**Affiliations:** 1 Geneva Cancer Registry, Global Health Institute, Geneva University, Geneva, Switzerland; 2 Cancer Survival Group, Faculty of Epidemiology and Population Health, Department of Non-Communicable Disease Epidemiology, London School of Hygiene & Tropical Medicine, London, UK

**Keywords:** Breast cancer, Prognostic factors, Treatment, Excess hazard model, Sensitivity analyses, Cancer registry

## Abstract

**Background:**

Breast cancer prognosis has dramatically improved over 40 years. There is, however, no proof of population ‘cure’. This research aimed to examine the pattern of long-term excess mortality due to breast cancer and evaluate its determinants in the context of cancer registry data.

**Methods:**

We used data from the Geneva Cancer Registry to identify women younger than 75 years diagnosed with invasive, localised and operated breast cancer between 1995 and 2002. Flexible modelling of excess mortality hazard, including time-dependent (TD) regression parameters, was used to estimate mortality related to breast cancer. We derived a single “final” model using a backward selection procedure and evaluated its stability through sensitivity analyses using a bootstrap technique.

**Results:**

We analysed data from 1574 breast cancer women including 351 deaths (22.3%). The model building strategy retained age at diagnosis (TD), tumour size and grade (TD), chemotherapy and hormonal treatment (TD) as prognostic factors, while the sensitivity analysis on bootstrap samples identified nodes involvement and hormone receptors (TD) as additional long-term prognostic factors but did not identify chemotherapy and hormonal treatment as important prognostic factors.

**Conclusions:**

Two main issues were observed when describing the determinants of long-term survival. First, the modelling strategy presented a lack of robustness, probably due to the limited number of events observed in our study. The second was the misspecification of the model, probably due to confounding by indication. Our results highlight the need for more detailed data and the use of causal inference methods.

## Background

Breast cancer is a major disease worldwide. Its prognosis has, however, improved rapidly during the last four decades [1–3]. Accordingly, there are increasing numbers of women who have survived breast cancer. Despite this, there is evidence for a lack of population ‘cure’, that is, the probability of dying as a consequence of the disease persists for many years after diagnosis [4, 5] even for women who were screen-detected [6].

The estimation of net survival has allowed these trends to be observed [7–9]. Unlike all other metrics, net survival evaluates the mortality arising only from the disease of interest, disregarding the influence of other causes of death [10]. In the context of long-term survival this is fundamental because the likelihood of death from other causes increases with follow-up time (i.e. with ageing of the patients). The use of net survival allows accurate comparisons of patient’s subgroups across space and time, between which mortality from other causes may vary considerably [9, 11].

Although there is a great interest, both clinically and epidemiologically, in the determinants of long-term survival for breast cancer patients, follow-up beyond 5 or 10 years has not been widely considered. The few studies with long-term observations have demonstrated that the associations of some covariables do vary with time since diagnosis in the long term [12], very few however considered very long-term follow-up and/or multiple covariables [13, 14]. In particular, the influence of treatment represents an interesting line of investigation since it is likely that certain treatments lead to severe long-term side effects [13, 15].

The Geneva Cancer Registry offers an ideal context to study the evaluation of determinants of long-term net survival. The cancer registry, initiated in 1970, allows long follow-up of cancer patients. The availability of detailed information for each woman’s tumour enables multivariable survival analysis.

In this research, we aim to evaluate long-term associations between prognostic factors and the excess mortality hazard for breast cancer patients diagnosed in Geneva, focusing especially on treatment variables. To reach this aim, we focus on early-stage tumours, which were surgically resected. We use flexible excess hazard regression models to account for potential time-varying and non-linear associations. We apply a systematic model selection process to build a “final” regression model, and check the stability of our “final” model by conducting a sensitivity analysis using bootstrap sampling.

## Methods

### Patient cohort

The Geneva Cancer Registry collects information on incident cancer cases from various sources, including hospitals, laboratories and private clinics, all of whom report newly diagnosed cancer cases. Trained registrars systematically extract information from the medical records and conduct further investigations in the case of missing data. The registry regularly estimates cancer patient survival, taking as the reference the date the diagnosis was confirmed or, if it preceded the diagnosis and was related to the disease, the date of hospitalisation. In addition to standard examination of death certificates and hospital records, patient’s vital status is assessed annually by matching the Registry’s database with information held by the Cantonal Population Office which maintains a live register of the resident population.

We included all women diagnosed with an invasive primary breast cancer in the Geneva Canton between 1995 and 2002. We restricted the sample to patients diagnosed with pathological TNM stage I and II disease who were treated with surgery (*N* = 2029). Among those patients, we excluded patients older than 75 years (*N* = 232). Information on stage was missing among only 60 (2.57%) patients with surgery. All women were followed up until 31st December 2013 (11 years of minimum follow-up).

### Ethical approval

The Geneva Cancer Registry has a general authorization to collect nominative data, and to analyze the anonymized data. Since the study did not require additional clinical information, approval of the Ethics Committee was not required.

### Prognostic factors and treatment

We focused on established prognostic factors and on treatment. *Age at diagnosis* (years) was included a priori as an irrefutable prognostic factor [16]. We considered *tumour size* (mm), *degree of differentiation* (Well vs. Moderately/Not differentiated), *nodal involvement* (No vs. Yes) and *hormone receptor status* (Negative vs. Positive) which together reflect the severity of the disease. We included *radiotherapy*, *chemotherapy* and *hormonal treatment* following surgery (each Yes vs. No, within 6 months after diagnosis) in order to examine the long-term associations of these systemic treatments with survival.

### Statistical modelling of the excess mortality hazard

We estimated the excess mortality hazard due to cancer for the patient group. The excess mortality hazard corresponds to the mortality hazard related only to the disease of interest (in our case, breast cancer) and is defined as the difference between the mortality observed amongst a cohort of patients and their expected (background) mortality [17, 18]. The association between covariables and excess mortality can vary with time since diagnosis, particularly when considering long-term follow-up. For example, a particular treatment might have a strong influence on excess mortality one year after diagnosis but a weaker influence at ten years (time-dependent, TD, association). Furthermore, continuous variables can display non-linear (NL) associations (for example, excess mortality might increase exponentially with age). We handled such complex associations through the flexible excess hazard model proposed by Charvat et al. [19], which follows the work of Remontet et al [20]. This excess hazard model is implemented in the “*mexhaz*” package written for R software [19, 21].

### Model building strategy

We applied the model building strategy suggested by Wynant and Abrahamowicz [22]. This iterative backward elimination procedure involves testing, for each variable, the presence of significant TD and, for continuous variables only, NL associations as well as the overall significance of the variable itself. An initial model including all variables, as well as all possible TD and NL associations, is fitted. Potentially spurious NL and TD associations are then eliminated one by one by using likelihood ratio tests and with a statistical threshold for significance of 0.05. Our initial model included:

- *age at diagnosis* (continuous, NL and TD associations included),
- *tumour size* (continuous, log-transformed, NL and TD associations included),
- *nodal involvement* (binary, TD association included, “Yes” as reference category),
- *grade of the tumour* (binary, TD association included, “Moderately/Not differentiated” as reference category),
- *hormone receptor status* (binary, TD association included, “Positive” as reference category),
- *radiotherapy* (binary, TD association included, “No” as reference category),
- *chemotherapy* (binary, TD association included, “No” as reference category) and
- *hormonal treatment* (binary, TD association included, “No” as reference category).

The model building strategy resulted in a single derived model (the “final” model), which included only those variables found to be significant, along with any significant TD and/or NL associations for these variables.

### Sensitivity analyses

We conducted a sensitivity analysis to examine the stability of the derived “final” model using a bootstrap technique [23]. This involved re-applying the model selection procedure to 300 random samples, drawn, with replacement, from the cohort of cancer patients. This procedure allows the evaluation of the strength of association between a particular covariable and the excess mortality hazard by the calculation of the bootstrap inclusion frequency (BIF) [23]. The BIF is the proportion of times a specific variable was included by the model selection process over the total number of bootstrap samples. We further considered only models where the association was plausible (outliers where estimated values of the Excess Hazard Ratio (EHR) were greater than 100 or less than 0.01 were excluded). We then plotted all the estimated functional forms of each covariable (*N* ≤ 300 due to convergence issues, see below), along with the averaged functional form calculated on all the retained samples.

## Results

### Patient cohort

The study included 1797 women diagnosed with first primary invasive breast cancer between 1995 and 2002 which was classified as stage I or II at diagnosis and treated surgically (Table 1). Data were missing for at least one co-variable for 12.4% of women. The highest proportion of missing data was for the size of the tumour (*N* = 72, 4.0%). Only women with complete data for all variables were considered for the modelling analyses (*N* = 1574, 87.6%) [24]. Of these, 351 died (22.3%) and 236 were censored (14.9%) before the end of follow-up. The median follow-up time was 12.8 years.

**Table 1:** Characteristics of the patients diagnosed with breast cancer between 1995 and 2002

	Radiotherapy				Chemotherapy				Hormonal treatment				Total	
	No		Yes		No		Yes		No		Yes			
	N	%	N	%	N	%	N	%	N	%	N	%	N	%
Age group														
< 40	16	5.5	71	4.7	14	1.4	73	9.2	39	7.9	48	3.7	87	4.8
40–49	79	27.1	280	18.6	125	12.4	234	29.6	149	30.3	210	16.1	359	20.0
50–59	78	26.8	574	38.1	348	34.6	304	38.4	158	32.1	494	37.9	652	36.3
60–69	87	29.9	448	29.7	374	37.2	161	20.4	114	23.2	421	32.3	535	29.8
70–79	31	10.7	133	8.8	145	14.4	19	2.4	32	6.5	132	10.1	164	9.1
Total	291	100	1506	100	1006	100	791	100	492	100	1305	100	1797	100
Size in mm														
0–9	44	15.1	227	15.1	222	22.1	49	6.2	84	17.1	187	14.3	271	15.1
10–19	91	31.3	709	47.1	512	50.9	288	36.4	172	35	628	48.1	800	44.5
20–29	66	22.7	329	21.8	170	16.9	225	28.4	120	24.4	275	21.1	395	22.0
30–39	45	15.5	115	7.6	56	5.6	104	13.1	51	10.4	109	8.4	160	8.9
40+	27	9.3	72	4.8	33	3.3	66	8.3	38	7.7	61	4.7	99	5.5
Missing	18	6.2	54	3.6	13	1.3	59	7.5	27	5.5	45	3.4	72	4.0
Total	291	100	1506	100	1006	100	791	100	492	100	1305	100	1797	100
Nodal involvement														
N+	82	28.2	418	27.8	127	12.6	373	47.2	137	27.8	363	27.8	500	27.8
N0	201	69.1	1064	70.7	863	85.8	402	50.8	351	71.3	914	70	1265	70.4
Missing	8	2.7	24	1.6	16	1.6	16	2	4	0.8	28	2.1	32	1.8
Total	291	100	1506	100	1006	100	791	100	492	100	1305	100	1797	100
Differentiation														
Well differentiated	186	63.9	927	61.6	497	49.4	616	77.9	346	70.3	767	58.8	1113	61.9
Moderately/ poorly differentiated	84	28.9	533	35.4	472	46.9	145	18.3	114	23.2	503	38.5	617	34.3
Missing	21	7.2	46	3.1	37	3.7	30	3.8	32	6.5	35	2.7	67	3.7
Total	291	100	1506	100	1006	100	791	100	492	100	1305	100	1797	100
Hormone receptors														
Positive	220	75.6	1300	86.3	920	91.5	600	75.9	253	51.4	1267	97.1	1520	84.6
Negative	38	13.1	174	11.6	45	4.5	167	21.1	194	39.4	18	1.4	212	11.8
Missing	33	11.3	32	2.1	41	4.1	24	3	45	9.1	20	1.5	65	3.6
Total	291	100	1506	100	1006	100	791	100	492	100	1305	100	1797	100
Complete data														
Complete	216	74.2	1358	90.2	900	89.5	674	85.2	397	80.7	1177	90.2	1574	87.6
Missing	75	25.8	148	9.8	106	10.5	117	14.8	95	19.3	128	9.8	223	12.4
Total	291	100	1506	100	1006	100	791	100	492	100	1305	100	1797	100

### “Final” derived model

The single derived model resulting from the model building strategy included associations with the excess mortality hazard which were linear and TD for *age*, linear and time-constant for *size of the tumour*, TD for *grade* of the tumour, time-constant for *chemotherapy* and TD for *hormonal treatment*. All the other variables *(nodal involvement, hormone receptor status and radiotherapy)* were considered not associated with the excess mortality hazard and were therefore excluded.

### Stability of the “final” derived model

The sensitivity analysis suggested that the model derived for the patient cohort was not very stable. For 60 out of 300 bootstrap samples (outliers included), the model did not reach convergence. The variables *size of tumour*, *hormone receptors status*, *age at diagnosis*, *grade* and *nodal involvement* displayed the highest BIFs in the sensitivity analysis (Table 2, more than 80%). However, not all of them were selected in the “final” model; here neither *nodal involvement* nor *hormone receptor status* showed evidence of an association with the excess mortality hazard. The covariables describing treatment were less frequently selected in the sensitivity analysis, with a BIFs of 75.4, 59.6 and 45.0% for *chemotherapy*, *radiotherapy* and *hormonal treatment* respectively, whilst in the “final” model, *chemotherapy* and a TD association for *hormonal treatment* were retained. Although TD associations were frequently observed in the sensitivity analysis for the covariables *hormone receptors status* and *age* (BIF 95.4 and 87.1% respectively), only the TD association for *age* was found to be significant in the “final” model. NL associations for *age* and *size of the tumour* were not retained in the “final” model, which was consistent with the low BIFs observed in the sensitivity analysis (18.8 and 34.2% respectively).

**Table 2:** Bootstrap Inclusion Frequency (BIF) for each co-variable and their type of associations following the sensitivity analysis

	BIF (%)		
	Main	Non-linear	Time dependent
Age	92.9	18.8	87.1
Size of the tumour	99.6	34.2	57.1
Nodal involvment	85.8	–	55.4
Grade of the tumour	90.8	–	51.7
Hormone receptors	97.1	–	95.4
Radiotherapy	59.6	–	18.8
Chemotherapy	75.4	–	32.9
Hormonal treatment	45	–	20.4

“-”: Not applicable

**Fig. 1:**
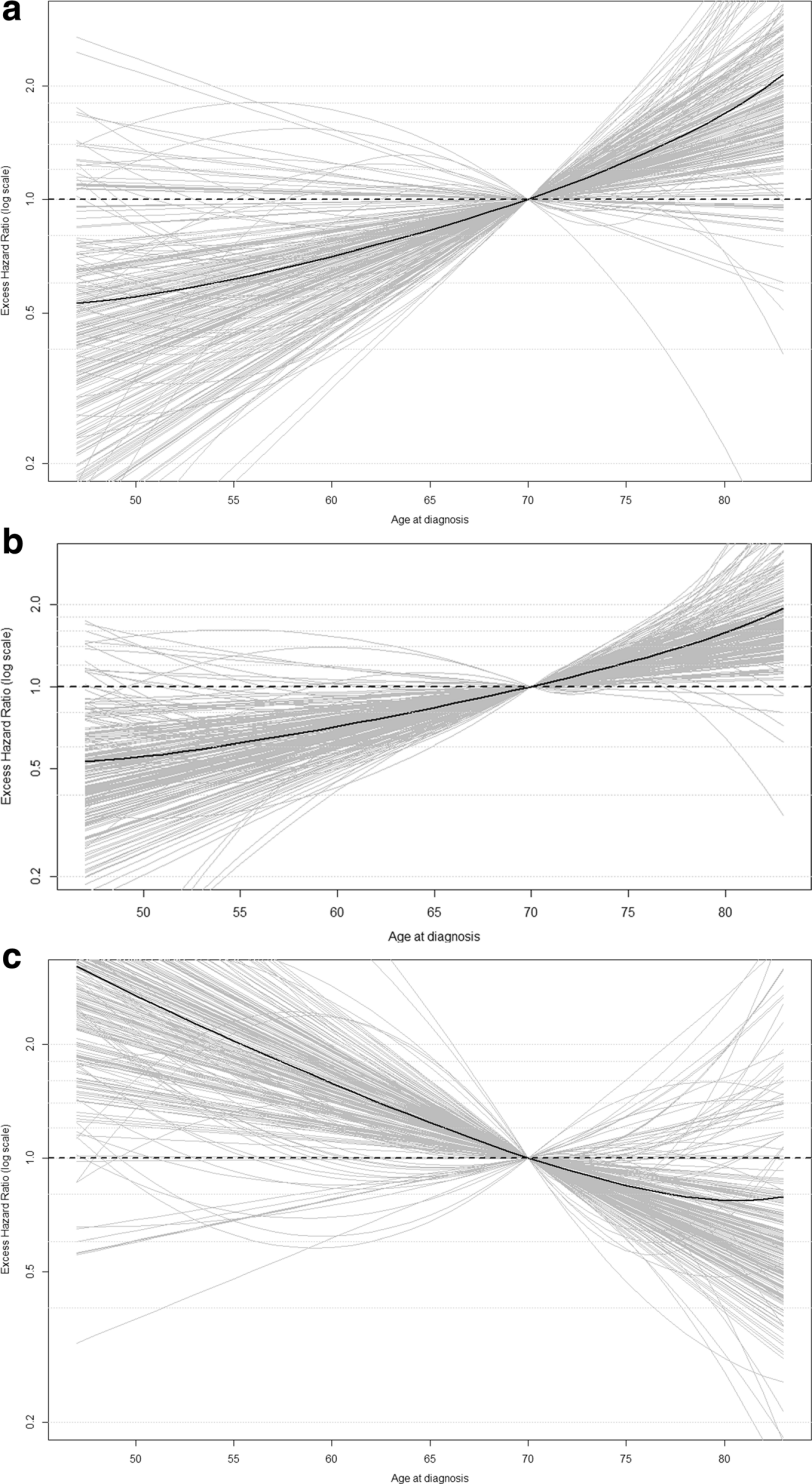
Excess hazard ratio for age at diagnosis, excluding outliers, using 70 years as the reference (**a**) 1 year after diagnosis. **b** 5 years after diagnosis. **c** 10 years after diagnosis

**Fig. 2:**
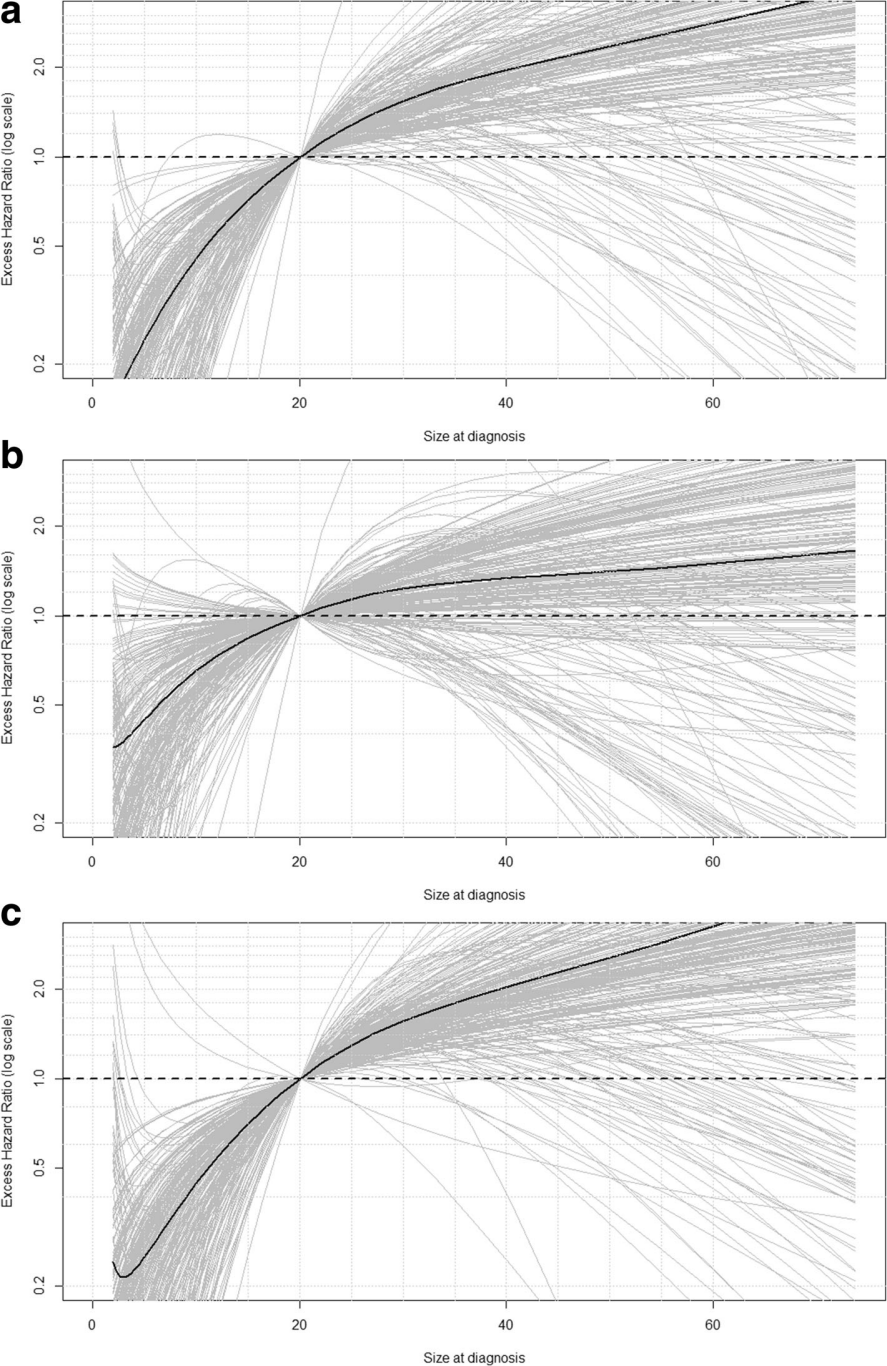
Excess hazard ratio for tumour size, excluding outliers, using 20 mm as a reference. **a** 1 year after diagnosis (**b**) 5 year after diagnosis (**c**) 10 year after diagnosis

**Fig. 3:**
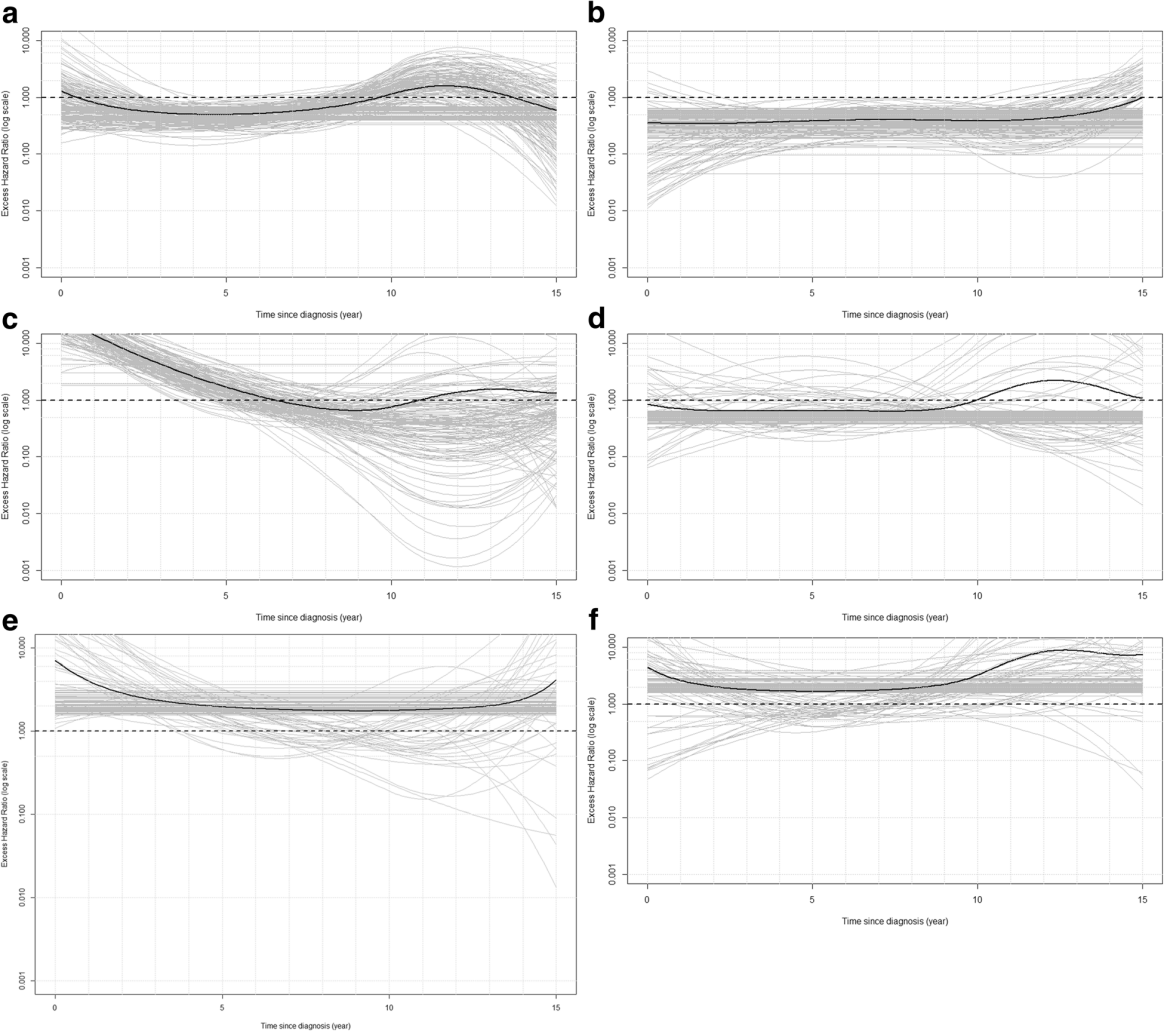
Excess hazard ratio for categorical covariables, excluding outliers. **a** Nodal involvement with Yes as reference category. **b** Grade with Moderately/Not differentiated as reference category. **c** Hormone receptor status with Positive as reference category. **d** Radiotherapy with No as reference category. **e** Chemotherapy with No as reference category. **f** Hormonal treatment with No as reference category

Figures 1, 2 and 3 display the associations between each of the covariables and the excess mortality hazard, as obtained from the sensitivity analysis (excluding outliers). The mean association across all samples (black solid line) is also displayed. These show that within the sensitivity analysis we observed a TD association for *age:* excess mortality increased with age during the first 10 years of follow-up (Fig. 1a-b) but reversed after this point (Fig. 1c). Figure 2 shows that excess mortality increased linearly with *tumour size* and that this association was constant over time since diagnosis. *Nodal involvement* was associated with higher excess mortality. There was evidence of a TD association for *hormone receptor status*, with negative receptors being associated with an increased risk of dying from breast cancer only during the first 5 years of follow-up. This was similar for *grade*: women with well differentiated tumours displayed a lower risk of dying from breast cancer, an association which also tended towards the null at the end of follow-up. *Radiotherapy* was associated with a decreasing risk of dying during the first 10 years after diagnosis, whereas receipt of *chemotherapy* and *hormonal treatment* were associated with an increasing risk during the entire follow-up period.

## Discussion

The determinants of long-term survival are currently of particular interest because of the dramatic increase in the number of patients surviving breast cancer matched to the observation that these women are never ‘cured’. Understanding the impact of prognostic factors and of treatment with time since diagnosis is therefore increasingly important. In this context, population-based data are crucial to understand how treatment influences the outcomes for all cancer patients. These aims should be distinguished from those of web-based models, which provide patients with an estimation of his/her survival according to his/her values of prognostic factors.

### Our approach

In order to estimate the long-term associations of prognostic factors and treatment with the risk of dying from breast cancer, we used observational data from the population-based Geneva Cancer Registry. For this purpose, we restricted our cohort to a relatively homogeneous group of younger patients (less than 75) with localised disease (stage I and II) and who had received surgery. The severity of the disease was controlled for through the combination of several covariables, and the analyses accounted for differences in individual characteristics. Furthermore, the estimation of the mortality related to the disease, after controlling for other causes, was based on flexible excess hazard regression models, which enable the assumptions of linear and time constant excess hazard ratios to be relaxed. Both of these assumptions are clinically unlikely in the context of long-term survival. We used a recommended strategy [22] for selection of covariables and their complex associations, and performed a sensitivity analysis to evaluate the reproducibility of the model [23].

Despite using a fairly homogeneous group of patients, this optimised and up-to-date modelling strategy, a clear process for variable and complex association selection and a sensitivity analysis, our results demonstrated a lack of stability and model misspecification, associated with unrealistic effects of some treatments (e.g. chemotherapy).

### Modelling issues

First, our sensitivity analysis demonstrated that the set of covariables included (eventually with NL and/or TD functional forms) in the “final” model for the excess mortality hazard was unstable. Because of this demonstrated instability, results obtained from a single model should be interpreted with caution. This is best illustrated by the fact that 20% of models did not reach convergence during the sensitivity analysis, as well as the fact that several variables selected for the single derived model were rarely retained in the sensitivity analysis (low BIF, e.g. TD for *hormonal treatment*). Meanwhile others not retained in the derived model were often selected by the sensitivity analysis (high BIF e.g. hormone receptors).

There are a number of possible reasons for this lack of robustness. The first is related to the context in which the study was conducted. Since breast cancer patients present with high survival, the number of events (“excess” death) is relatively low in breast cancer data, even where long-term follow-up is available. This is especially true for the fairly small Geneva population (495,000 inhabitants) and for the study population which was restricted to early-stage cancer patients. It is recommended that at least 10 events per parameter should be included when estimating regression coefficients [25, 26]. Because we considered both time-dependent and non-linear associations for all prognostic variables, the number of parameters included in our model was large relative to the number of deaths. The convergence issues that we encountered are therefore likely to be explained, in part, by a lack of information from the observed data. However, decreasing the number of parameters (either by reducing the number of variables, or excluding some complex associations) would not have been a better strategy, given that our core aim was to try to better understand the long-term associations of prognostic covariables for breast cancer patients. Neither was it practical to increase the number of women in order to increase the number of events since this could only have been done by including women with advanced disease (for which treatment protocols are very different) or by including elderly women (who do not have the opportunity for long-term follow-up, and for whom the excess regression modelling would not make sense on the longer term [10].

The analysis excluded 12.3% of the cohort because of missing data, thus leading to a loss of information. However this proportion is relatively low for these types of observational data and complete-case analyses have been proved to be sufficiently efficient for such ranges of missing data proportion [24]. Also, our aim was to highlight the difficulties encountered with modelling in the context of observational data. We therefore performed a complete-case analysis in order not to dilute the message with issues related to multiple imputation.

It is possible that the lack of stability may have been a result of the modelling approach. We consider this unlikely, however. The flexible regression model we applied has been purposefully designed to estimate excess mortality hazard [19] and take into account complex associations. The model selection strategy has previously been shown to be efficient and successful in detecting the correct complex associations as well as eliminating spurious ones [22].

The second main issue was that our strategy was unable to fully control for confounding by indication leading to model misspecification. This would be an issue even with a perfectly robust model. This confounding is best illustrated by the unexpected results for chemotherapy and hormonal treatment. Women receiving these treatments experienced an increased risk of dying from breast cancer compared to women who did not receive them (Fig. 3). This reflects the fact that the patients in the cohort who received chemotherapy and hormonal treatment were probably those with more advanced disease at diagnosis, among the early-stage cases (Table 1). This represents a limitation of our strategy, which was not able to account for the fact that almost all women who were likely to benefit from these therapies were given them, resulting in a sparse comparison group within the patient cohort (confounding by indication). We performed a stratified analysis to explore this (data not shown). We grouped patients with very similar characteristics together and compared their survival according to receipt of chemotherapy or not. This similarly showed an increased risk in the excess hazard of death associated with chemotherapy. This strongly suggests that additional information about the prognosis of patients not receiving chemotherapy is missing from our dataset, and that this led to misspecification of the model.

In addition, interactions between treatment received and other co-variables might be required. Although we planned to examine the existence of such interactions, they were tricky to implement due to the convergence issues we encountered during the modelling process, and not reasonable to explore in our small sample size dataset.

### Other possible strategies

Our results point towards the need for different statistical strategies in addition to our modelling strategy to be better able to examine these effects more than only the associations. Causal inference analyses would be one suitable approach [27–29]. The objective of causal inference is to mimic the randomised trial that would have been set for the research question by using observational data and specific statistical techniques. Propensity score methods could, for example, be implemented within the flexible regression models we have used here [30, 31]. In our work, we assumed that people were treated at the date of diagnosis, which is probably not correct for all patients. Also, some changes in the prognostic factors values for some patients (e.g. growth of the tumor size) may suggest that a treatment needs to be undertaken later on after the diagnosis. In the presence of such time-varying confounding, other approaches such as parametric g-formula [32], structural nested models or marginal structural models with inverse probability weighting would also be of interest, especially for the long-term treatment effect [33, 34] . All these approaches assume the models to be well specified, which is not so easy to achieve. Various approaches, including using machine learning techniques, have been developed to minimise model misspecification [35]. This would however require much more detailed data, including comorbidities and other factors used to define the treatment choice. Furthermore, software to implement causal inference techniques is not yet available for the excess mortality hazard. Further methodological research is thus required to enable such analyses to be conducted.

### Clinical interpretations

Nevertheless, a few cautious clinical interpretations can be drawn from these data. Some co-variables presented high BIFs within the sensitivity analysis and the observed associations appeared stable to the exclusion of outliers suggesting that they are indicative of a robust, underlying associations. Consistent with Jatoi et al. [14] we found that patients with negative hormone receptors presented a higher excess mortality during the first years after diagnosis compared to those who have positive hormone receptors (BIF 95.4%). Regarding age at diagnosis, our results matched those found by Cluze et al. [16] which showed the risk of dying from breast cancer was associated with increasing age at 1 and 5 years after diagnosis but that this association reversed at 10 years (BIF 87.1%). In addition to hormone receptor status and age at diagnosis, tumour size, grade and nodal involvement displayed associations which were similar to those described in a previous meta-analysis [12]. Although our results are broadly consistent with previous studies, caution should be exercised in reporting the size of these associations, given that they have been derived from models, which display a lack of robustness. We observed a time-dependent association for radiotherapy: patients treated with radiotherapy exhibited a decreased excess mortality hazard in the first 10 years following their diagnosis but an increased hazard afterwards. This association was, however, sensitive to the inclusion or exclusion of outliers. That said, it could potentially correspond to late side effects of treatment, in particular cardiac complications, which are known as a likely consequence of irradiations given close to the heart [36–38].

## Conclusion

Our research aimed to estimate the long-term associations of prognostic factors and treatment for breast cancer using flexible excess hazard-based regression models for patients diagnosed in Geneva between 1995 and 2002. Our study highlights the challenges of interpreting these associations in observational data and as well as the need for high quality and detailed clinical information at a population level so that these associations can be examined in detail. With such data, causal inference methods could be applied to be able to describe an effect rather than an association. However, applying causal inference methods requires further methodological work and the development of specialist software for the use of causal inference in the context of excess hazard modelling.
